# 848. Approaches to Optimize Recruitment of Historically Underrepresented Black and Hispanic/LatinX MSM, Transgender, and Gender Non-binary Individuals into the Lenacapavir for PrEP (PURPOSE 2) Trial

**DOI:** 10.1093/ofid/ofab466.1043

**Published:** 2021-12-04

**Authors:** Michelle Cespedes, Jill Blumenthal, Karam Mounzer, Moti Ramgopal, Theo Hodge, Ayana Elliott, A C Demidont, C Chauncey Watson, Christoph C Carter, Alex Kintu, Moupali Das, Jared Baeten, Onyema Ogbuagu

**Affiliations:** 1 Icahn School of Medicine at Mount Sinai, New York, NY; 2 UC San Diego Health, San Diego, CA; 3 Philadelphia FIGHT, Philadelphia, PA; 4 Midway Research Center, Ft. Pierce, FL; 5 Washington Health Institute, Washington, DC; 6 Gilead Sciences Inc., Foster City, CA; 7 Yale University, New Haven, CT

## Abstract

**Background:**

Black and Hispanic/Latinx gay and other men who have sex with men (MSM), transgender women (TGW), transgender men (TGM), and gender nonbinary individuals (GNB) have been historically underrepresented in HIV prevention trials despite being disproportionately affected by the disease. Therefore, studies of pre-exposure prophylaxis (PrEP), a highly effective intervention for reducing HIV incidence, should include these individuals, and doing so would promote generalizability of the findings.

**Methods:**

PURPOSE 2 (GS-US-528-9023) will evaluate a twice-yearly long-acting subcutaneous, first in class capsid inhibitor, lenacapavir, for PrEP in MSM, TGW, TGM, and GNB in the US, Brazil, Peru, and South Africa. The study team adopted a multifactorial approach to address historic underrepresentation. This included a literature review to assess successful evidence-based approaches for increasing enrollment of Black and Hispanic/ LatinX MSM, TG, and GNB individuals. We engaged with community and patient advocates as well as key stakeholders to solicit feedback prior to protocol development.

**Results:**

We established a trial-specific Global Community Advisory Group and implemented their recommendations for site selection, investigator and staff diversity, and strong linkage with community-based organizations. We recruited new community-based research sites and principal investigators (PIs) to mirror historically underrepresented populations and emphasized mentorship of junior sub-Is by seasoned PIs to support enrollment and retention. We developed required trainings for all study and site staff on good participatory practices for PrEP, anti-racism and transgender cultural humility. We established recruitment goals of 50% Black and 20% Hispanic/LatinX MSM in the US, and 20% TGW study-wide. Our strategy to ensure achievement of these overall goals involves nuanced site-specific recruitment goals considering site capacity, local demographics, and HIV incidence data. We will review metrics weekly during enrollment and make any necessary adjustments.

**Conclusion:**

Using novel approaches, we have carefully chosen with whom, where, and how we will collaborate to increase the diversity, equity, and inclusion in the PURPOSE 2 trial.

**Disclosures:**

**Michelle Cespedes, MD, MS**, **Gilead Sciences Inc.** (Scientific Research Study Investigator, Advisor or Review Panel member, Research Grant or Support)**GlaxoSmithKline** (Scientific Research Study Investigator, Research Grant or Support) **Jill Blumenthal, MD**, **Gilead Sciences** (Grant/Research Support, Scientific Research Study Investigator) **Karam Mounzer, MD**, **Epividian** (Advisor or Review Panel member)**Gilead Sciences Inc.** (Consultant, Scientific Research Study Investigator, Research Grant or Support, Speaker’s Bureau)**Janssen** (Consultant, Research Grant or Support, Speaker’s Bureau)**Merck** (Research Grant or Support, Speaker’s Bureau)**ViiV Healthcare** (Consultant, Speaker’s Bureau) **Moti Ramgopal, MD FACP FIDSA**, **Abbvie** (Scientific Research Study Investigator, Speaker’s Bureau)**Gilead** (Consultant, Scientific Research Study Investigator, Speaker’s Bureau)**Janssen** (Consultant, Scientific Research Study Investigator, Research Grant or Support, Speaker’s Bureau)**Merck** (Consultant, Scientific Research Study Investigator)**ViiV** (Consultant, Scientific Research Study Investigator, Speaker’s Bureau) **Ayana Elliott, DNP, APRN, FNP-C, NEA-BC**, **Gilead Sciences Inc.** (Employee, Shareholder) **A.C. Demidont, MD**, **Gilead Sciences Inc.** (Employee, Shareholder) **C. Chauncey Watson, MD**, **Gilead Sciences Inc.** (Employee, Shareholder) **Christoph C. Carter, MD**, **Gilead Sciences Inc.** (Employee, Shareholder) **Alex Kintu, MD, ScD**, **Gilead Sciences Inc.** (Employee, Shareholder) **Moupali Das, MD**, **Gilead Sciences Inc.** (Employee, Shareholder) **Jared Baeten, MD, PHD**, **Gilead Sciences Inc.** (Employee, Shareholder) **Onyema Ogbuagu, MD**, **Gilead Sciences Inc.** (Scientific Research Study Investigator, Advisor or Review Panel member, Research Grant or Support, Other Financial or Material Support)**ViiV Healthcare** (Advisor or Review Panel member)

